# Subclinical Cerebral Lesions in Migraine: A Proof‐of‐Concept Study on Platelet Aggregation

**DOI:** 10.1111/ene.70432

**Published:** 2026-11-02

**Authors:** Ilaria Maestrini, Mariaignazia Curreli, Marta Puma, Costanza Giannì, Eleonora Schiera, Giada Giuliani, Massimiliano Toscano, Marta Altieri, Patrizia Pantano, Fabio Pulcinelli, Vittorio Di Piero

**Affiliations:** ^1^ Department of Systems Medicine University of Rome Tor Vergata Rome Italy; ^2^ Department of Human Neurosciences Sapienza University of Rome Rome Italy; ^3^ Department of Experimental Medicine Sapienza University of Rome Rome Italy; ^4^ Department of Neurology Sant'Antonio Abate Hospital Trapani Italy; ^5^ IRCCS Neuromed Pozzilli Italy; ^6^ Department of Neurology Fatebenefratelli Hospital—Gemelli Isola Rome Italy

**Keywords:** cerebral white matter lesions, ischaemia, migraine, platelet aggregation, subclinical cerebral lesions

## Abstract

**Background:**

Migraine patients exhibit cerebral white matter lesions (WMLs) more frequently than the general population, with a potential micro‐ischemic origin. Although platelet function in migraine patients has been extensively studied, no previous research has evaluated platelet function in those with WMLs. Our aim was to assess platelet aggregation in migraine patients with WMLs, to investigate their potential correlation with ischemic pathophysiology.

**Methods:**

We enrolled consecutive 34 patients diagnosed with episodic or chronic migraine, with or without aura (ICHD3 criteria), out of attacks, including 20 with WMLs and 14 without lesions. Patients on antiplatelet therapy, or those with inherited platelet disorders or thrombophilia, were excluded. All patients underwent MRI to identify and quantify WMLs in mm^3^. Platelet aggregation was assessed in response to various agonists (ADP, collagen, epinephrine, and arachidonic acid) and compared to a group of matched healthy volunteers (HV).

**Results:**

Platelet aggregation in response to epinephrine was lower in migraine patients compared to HV. Among migraine patients, those with a lesion load ≥ 146.25 mm^3^ (forth quartile of the WMLs total volume) showed significantly higher platelet aggregation in response to epinephrine (1.25 and 2.5 μM), compared to those with a lesion load < 146.25 mm^3^ or no lesions (*p* = 0.043 and *p* = 0.0047, respectively).

**Conclusions:**

Migraine is associated with reduced platelet aggregation compared to HV, which may serve as a protective mechanism. However, in patients with a higher burden of WMLs, platelet aggregation levels were comparable to those of HV, raising the possibility of an ischemic contribution to WMLs in migraine patients.

## Graphical abstract

We assessed platelet aggregation in patients with migraine (*n* = 34) with or without cerebral white‐matter lesions (WMLs) versus matched controls (*n* = 34), with WMLs volume quantified by MRI (mm^3^). Aggregation to ADP, collagen, epinephrine, and arachidonic acid was measured; epinephrine‐induced aggregation at 1.25–2.5 μM was overall lower in migraine than controls, but was higher in migraine patients with WMLs burden ≥ 146.25 mm^3^ (*p* = 0.043 and 0.0047). These findings suggest that platelet hyporeactivity may be protective in migraine, whereas increased aggregation with higher WMLs burden raises the possibility of an ischaemic contribution to WMLs in migraine.

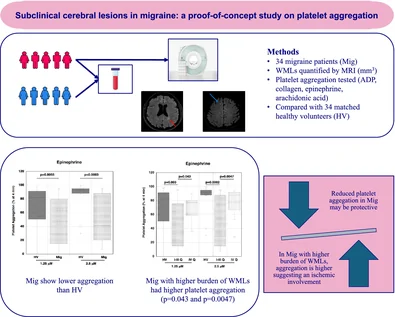

**Abbreviations**
ADP: adenosine diphosphate
DWMLs: deep white matter lesions
HV: healthy volunteers
IQR: interquartile range
MA: migraine with aura
MI: myocardial infarction
MO: migraine without aura
MRI: magnetic resonance imaging
OR: odds ratio
PFO: patent foramen ovale
PRP: platelet‐rich plasma
PVWMLs: periventricular white matter lesions
WMLs: white matter lesions

## 1 Introduction

Migraine and stroke are among the most prevalent and disabling diseases globally, ranking highly in terms of disease burden within the 10–49 age group for migraine and across all age groups for stroke, according to the Global Burden of Diseases, Injuries, and Risk Factors Study 2019 [1]. Epidemiological research has suggested a potential association between migraine and stroke [2, 3]. More recent meta‐analytic and genetic evidence suggests that migraine with aura (MA)—but not migraine without aura (MO)—is independently associated with approximately a two‐fold increased risk of ischaemic stroke, underscoring the importance of differentiating migraine subtypes when considering cerebrovascular risk [4, 5, 6]. Given the lower incidence of conventional vascular risk factors in patients with ischaemic stroke who also suffer from migraine, the role of migraine‐related mechanisms becomes increasingly important in the pathogenesis of ischaemic damage. Among these, the roles of patent foramen ovale (PFO) and MA have been increasingly recognized, especially in relation to cryptogenic stroke and sex‐specific susceptibility profiles [7].

Neuroimaging studies have indicated a higher incidence of infarct‐like lesions and white matter lesions (WMLs) in individuals with migraine compared to the general population, implying an increased cerebral vulnerability to ischemia in migraine‐susceptible brains [8, 9, 10]. The CAMERA (Cerebral Abnormalities in Migraine, an Epidemiological Risk Analysis) study, which investigated a population‐based sample of Dutch adults aged 30–60, found a trend toward a higher incidence of deep WMLs and subclinical infarcts in migraine patients compared to controls [8, 9]. These infarct‐like lesions were predominantly located in the cerebellar region of the posterior circulation and were more frequently observed in patients with MA than in those with MO (8.1% vs. 2.2%, *p* = 0.03) with an adjusted odds ratio (adjOR) of 13.7 (95% CI 1.7–112). Additionally, those with a high frequency of attacks (one or more per month) showed an odds ratio (OR) of 15.8 (95% CI 1.8–140) [8, 9]. Notably, the follow‐up study conducted 9 years later (CAMERA‐2 study) found that women with migraine experienced a progression of deep WMLs, suggesting an anatomically progressive disease, although there was no significant increase in infarct‐like lesions in the posterior circulation [11]. Similarly, the Epidemiology of Vascular Aging study, which examined a population‐based cohort of 780 elderly participants (mean age 69 years), also found a strong association between MA and the cumulative volume of deep WMLs and brain infarcts [12].

However, not all studies consistently demonstrate an increased risk of subclinical infarcts in migraine patients. The discrepancies may be attributed to differences in study design, participant characteristics (age, gender, headache or aura frequency, and conventional vascular risk factors), diagnostic assessments, and differences in neuroimaging protocols, including magnetic field strength, scanner type, sequence selection, or slice thickness [13].

The mechanisms by which migraine might increase the risk of cardiovascular disease are likely multifactorial. Although definitive evidence explaining the pathophysiological link between ischemic stroke, myocardial infarction (MI), and migraine is lacking, increased platelet activation and aggregation represent a plausible unifying theory [14].

The precise etiology of cerebral lesions in migraine patients remains unknown, though it is hypothesized that these lesions may be micro‐ischemic in nature. While platelet function has been studied extensively in migraine patients [15, 16, 17, 18, 19, 20], no study has yet evaluated platelet function in migraine patients with brain lesions. Our objective was to evaluate platelet aggregation in migraine patients with WMLs to investigate whether these brain lesions could be linked to an ischemic mechanism.

## 2 Methods

### 2.1 Population

We enrolled consecutive patients diagnosed with episodic or chronic migraine, with or without aura (ICHD3 criteria), at the tertiary Headache Centre of the ‘Sapienza’ University of Rome. Patients were enrolled during the interictal period (i.e., at least 3 days after and before the last attack) for episodic migraine patients and at least 24 h after the intake of symptomatic pain therapy. Patients with medication‐overuse headache were excluded from the study. Additional exclusion criteria included: (i) use of medications known to affect platelet function (aspirin, ticlopidine, clopidogrel, cilostazol, dipyridamole), or use of oral anticoagulants or heparin; (ii) congenital and/or acquired platelet function disorders; (iii) thrombophilia; (iv) a history of established cardiovascular diseases including ischemic heart disease (e.g., MI, angina, or acute coronary syndrome), cerebrovascular disease (e.g., stroke or transient ischemic attack), peripheral vascular disease, moderate to severe heart failure, or diabetes; and (v) the presence of inflammatory or autoimmune diseases.

All patients underwent a structured interview to document current and past headache characteristics and medical history, including cardiovascular risk factors. Each patient underwent a general and neurological physical examination, and the presence of a right‐to‐left shunt was assessed via contrast‐enhanced transcranial Doppler ultrasound to rule out a patent foramen ovale (PFO). Patients exhibiting any evidence of a right‐to‐left shunt were excluded from the study.

All patients underwent standardized clinical and laboratory screening as part of the routine outpatient evaluation. A comprehensive thrombophilia panel was performed to exclude inherited or acquired prothrombotic conditions. This included testing for lupus anticoagulant, anticardiolipin and anti‐β2‐glycoprotein I antibodies, protein C, protein S, antithrombin, homocysteine, and factor V/II mutations. Autoimmune screening included antinuclear antibodies, extractable nuclear antigen panel, anti‐dsDNA, rheumatoid factor, C‐reactive protein, and erythrocyte sedimentation rate. Medical records were also reviewed to rule out systemic autoimmune diseases. In addition, all patients underwent carotid Doppler ultrasound and comprehensive cardiac evaluation, including transthoracic echocardiography and 24‐h Holter ECG monitoring.

Thirty‐four headache‐free healthy volunteers (HV) were enrolled from hospital staff during the same study period as the migraine cohort. Eligibility was confirmed by a structured interview that excluded regular medication use and any cardiovascular, neurological, metabolic, autoimmune, or inflammatory disorders that could confound platelet assays. Controls were individually pair‐matched (1:1) to patients with migraine on sex and exact age, yielding no residual age variability; when multiple candidates met these criteria, the volunteer with the closest body‐mass index was selected, yielding two cohorts that were closely comparable across demographic variables.

### 2.2 Neuroradiological Assessment

All participants underwent a 3‐Tesla brain MRI (Verio, Siemens AG, Erlangen, Germany) with a standardized protocol using a 12‐channel head coil designed for parallel imaging (GRAPPA, Generalized Autocalibrating Partially Parallel Acquisitions). The MRI sequences included: (1) 3D T1‐weighted MPRAGE (magnetization‐prepared rapid acquisition with gradient echo), (2) double turbo spin‐echo (T2‐weighted and proton density‐weighted images), and (3) high‐resolution 3D fluid‐attenuated inversion recovery (3D‐FLAIR).

A neuroradiologist (C.G.), blinded to migraine diagnosis and clinical data, assessed infarcts and WMLs. Infarcts were defined as non‐mass parenchymal defects within a vascular distribution, isointense to cerebrospinal fluid on all sequences, and, when supratentorial, surrounded by a hyperintense rim on FLAIR and proton density images, according to the CAMERA study [21]. Infarcts were recorded based on number, location, and size. Virchow‐Robin spaces were excluded as infarcts based on location, shape, size, and absence of a hyperintense border. Infarcts were categorized by vascular territory using topographical maps: anterior/carotid circulation, posterior circulation, basal ganglia, and corona radiata/centrum semiovale.

WMLs were segmented using semi‐automated software (Jim v.5.2) and classified as periventricular (PVWMLs) or deep (DWMLs). White matter lesions had to be hyperintense on all sequences. Areas of hyperintensity were detected and segmented on DP‐weighted images and considered as WMLs. T2‐weighted images and 3D‐FLAIR images were used as references (Figures S1 and S2). The total volume of WMLs for each patient was calculated in cubic millimeters (mm^3^) based on the segmentation mask.

No radiological evidence of multiple sclerosis or other inflammatory white matter disease was found.

### 2.3 Platelet Aggregation

Platelet aggregation was performed in patients with migraine compared to a group of HV matched for age and gender as previously reported [22]. Briefly, blood samples were drawn from fasting patients and during the interictal period for patients with episodic migraine. Non‐Steroidal Anti‐Inflammatory Drugs or other medications affecting platelet function were discontinued 24 h before analysis. Blood samples were collected in 3.8% sodium citrate tubes for platelet aggregation and in an EDTA tube for platelet count. Platelet‐rich plasma (PRP) was obtained as previously reported [23]. Briefly citrate venous blood was collected from the donors. Blood samples were centrifuged at 200 g for 15 min and platelet‐rich plasma (PRP) was collected; it was then centrifuged at 2000 *g* for 10 min for platelet‐poor plasma preparation.

Platelet aggregation was evaluated in PRP, in a four‐channel aggregometer (AggRam, Helena Laboratories, Beaumont, TX, USA), according to Born's method [24]. Aggregation was performed in PRP activated with the following agonists: (i) ADP (1.25 and 2.5 μM; Helena Bioscience, Tyne and Wear, UK), (ii) epinephrine (1.25 and 2.5 μM; Helena Bioscience, Tyne and Wear, UK), (iii) collagen (1.25 and 2.5 μg/mL; Mascia Brunelli Milan, Italy); and arachidonic acid (0.5 mM; Helena Bioscience, Tyne and Wear, UK).

### 2.4 Statistics

Quantitative variables are expressed as means (± standard deviation) for normally distributed data or medians (interquartile range) otherwise. Categorical variables are expressed as percentages. Normality of the distributions was assessed using histograms and the Shapiro–Wilk test. Group comparisons for categorical variables were performed using the *χ* ^2^ test with Yates correction or Fisher's exact test as appropriate. Continuous variables were compared using the Mann–Whitney *U*‐test. The platelet aggregation results are shown as a BOX plot graph, which shows the distribution around the median of the population. Results are reported as median, minimum value (min.), and maximum value (max.). The data were analyzed using the software Statistical Package for the Social Sciences 26 for Windows (IBM Corp, Armonk, USA) and KaleidaGraph Software 3.6. Differences were calculated using Wilcoxon's test for unpaired data, a non‐parametric rank test. A *p*‐value < 0.05 was considered statistically significant.

## 3 Results

We enrolled 34 patients, median age 42.5 years (IQR 33–51), 28 women (82.4%). Four patients (11.8%) had two risk factors, and seven (20.6%) had one mild, well‐controlled vascular risk factor (such as arterial hypertension, hypercholesterolaemia, hypertriglyceridemia, or light smoking). The remaining 23 patients (67.7%) had no risk factors. The median history of migraine was 25.5 years; around one third (35%) of patients presented with chronic migraine and almost two thirds (64.7%) of patients had migraine with aura, a prevalence higher than that reported in the general population. The frequency of migraine was intermediate with a median of 5 headache days per month (IQR 2–11) and only 23.5% of patients underwent prophylactic therapy at the time of enrolment. The clinical characteristics of the study population are reported in Tables 1 and 2.

**TABLE 1:** Bivariate comparison of demographic characteristics and vascular risk factors in patients with and without white matter lesions (WMLs).

	All (*n* = 34)	WMLs		*p value*
		Present (*n* = 20)	Absent (*n* = 14)	
*Demographic characteristics*				
Age, y, median (IQR)	42.5 (33–51)	41.5 (37–51)	43 (32–52)	0.727
Female	28 (82.4)	18 (90.0)	10 (71.4)	0.173
Body mass index, kg/m^2^, median (IQR)	23 (20.7–27.5)	23 (21–27)	23.3 (19.7–28)	0.820
*Vascular risk factors*				
Arterial hypertension	5 (14.7)	4 (20.0)	1 (7.1)	0.299
Hypercholesterolemia	5 (14.7)	4 (20.0)	1 (7.1)	0.299
Hypertriglyceridemia	2 (5.9)	1 (5.0)	1 (7.1)	0.661
Smoking habit	7 (20.6)	4 (20.0)	3 (21.4)	0.622
Alcohol abuse	—	—	—	—
Physical activity	23 (67.3)	14 (70.0)	9 (64.3)	0.505
Estroprogestinic therapy	1 (2.9)	1 (5.0)	0 (0.0)	0.588
Menopause	8 (23.5)	6 (30.0)	2 (14.3)	0.261

*Note:* Values are number (%) of patients, unless otherwise indicated.

**TABLE 2:** Bivariate comparison of migraine characteristics in patients with and without white matter lesions (WMLs).

	All (*n* = 34)	WMLs		*p value*
		Present (*n* = 20)	Absent (*n* = 14)	
Age of onset, median (IQR)	16 (10–21)	16.5 (7.5–24)	15.5 (10–21.5)	0.635
Years of migraine, median (IQR)	25.5 (13–33)	29.5 (12–34.5)	25 (14–32)	0.727
Headache days/month, median (IQR)	5 (2–11)	3 (2–13.2)	5.5 (3–15)	0.603
Chronic migraine	12 (35)	8 (40.0)	4 (28.6)	0.377
History chronic migraine	15 (44.1)	10 (50.0)	5 (35.7)	0.409
Migraine with aura	22 (64.7)	12 (60.0)	10 (71.4)	0.377
Ongoing prophylactic therapy	8 (23.5)	4 (20.0)	4 (28.6)	0.428
*Topiramate*	2 (5.9)	2 (10.0%)	—	
*β‐blockers*	1 (2.9)	—	1 (7.1)	
*Amitriptyline*	4 (11.8)	2 (10.0)	2 (14.3)	
*Calcium antagonists*	1 (2.9)	—	1 (7.1)	
Nb. previous prophylactic therapies, median (IQR)	1 (0–2)	1 (0–3)	1 (0–1)	0.131

*Note:* Values are number (%) of patients, unless otherwise indicated.

Out of 34 patients, 20 patients (58.8%) presented white matter lesions (WMLs), of whom 9 patients (45%) had PVWMLs, and 19 (95%) had DWMLs. None presented lacunar infarcts.

Patients with WMLs did not significantly differ from patients without WMLs for demographic characteristics and vascular risk factors (Table 1) as well as for migraine characteristics (Table 2).

The median lesion volume was 38 mm^3^ [IQR 0–146.25]. The lesion volume was significantly higher in patients with MO compared to patients with MA (median volume 185 [IQR 0–427] vs. 16 [0–106], *p* = 0.036, respectively).

Patients with migraine and HV were matched on sex and identical in age (SD of pairwise age differences = 0); accordingly, no inferential tests were applied to these prespecified matching variables.

We found that platelet aggregation in response to epinephrine at 1.25 and 2.5 μM was lower in migraine patients compared to healthy volunteers (*p* = 0.0055 and *p* = 0.0003, respectively) (Figure 1). Among patients with migraine, patients who had a lesion load ≥ 146.25 mm^3^ (fourth quartile of the WMLs total volume), presented a higher platelet aggregation induced by epinephrine at 1.25 and 2.5 μM than those with a lesion load < 146.25 mm^3^ or no lesions (first to third quartile) (*p* = 0.043 and *p* = 0.0047, respectively) (Figure 2). Moreover, patients with a lesion load < 146.25 mm^3^ or no lesion (I–III quartile), presented a lower platelet aggregation induced by epinephrine at 1.25 and 2.5 μM than HV (*p* = 0.003 and *p* = 0.0002, respectively) (Figure 2).

**FIGURE 1:**
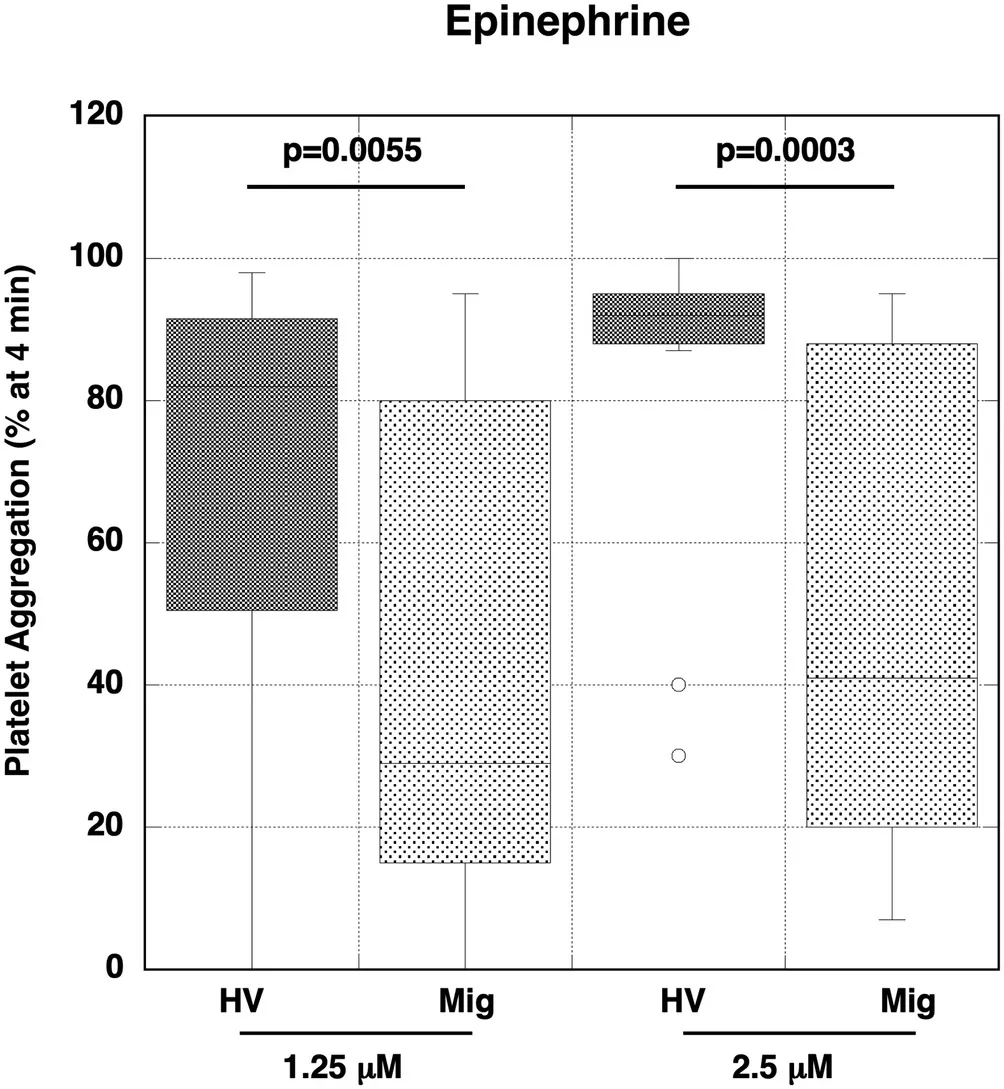
Assessment of platelet aggregation induced by epinephrine between healthy volunteers and patients with migraine. On the left side, the comparison of platelet aggregation induced by epinephrine at the concentration of 1.25 μM between healthy volunteers (HV—dark gray box) and patients with migraine (Mig—light gray box). On the right side, the comparison of platelet aggregation induced by epinephrine at the concentration of 2.5 μM between healthy volunteers (HV—dark gray box) and patients with migraine (Mig—light gray box). The median value and 25th to 75th percentiles are reported. *p* values are reported when significant.

**FIGURE 2:**
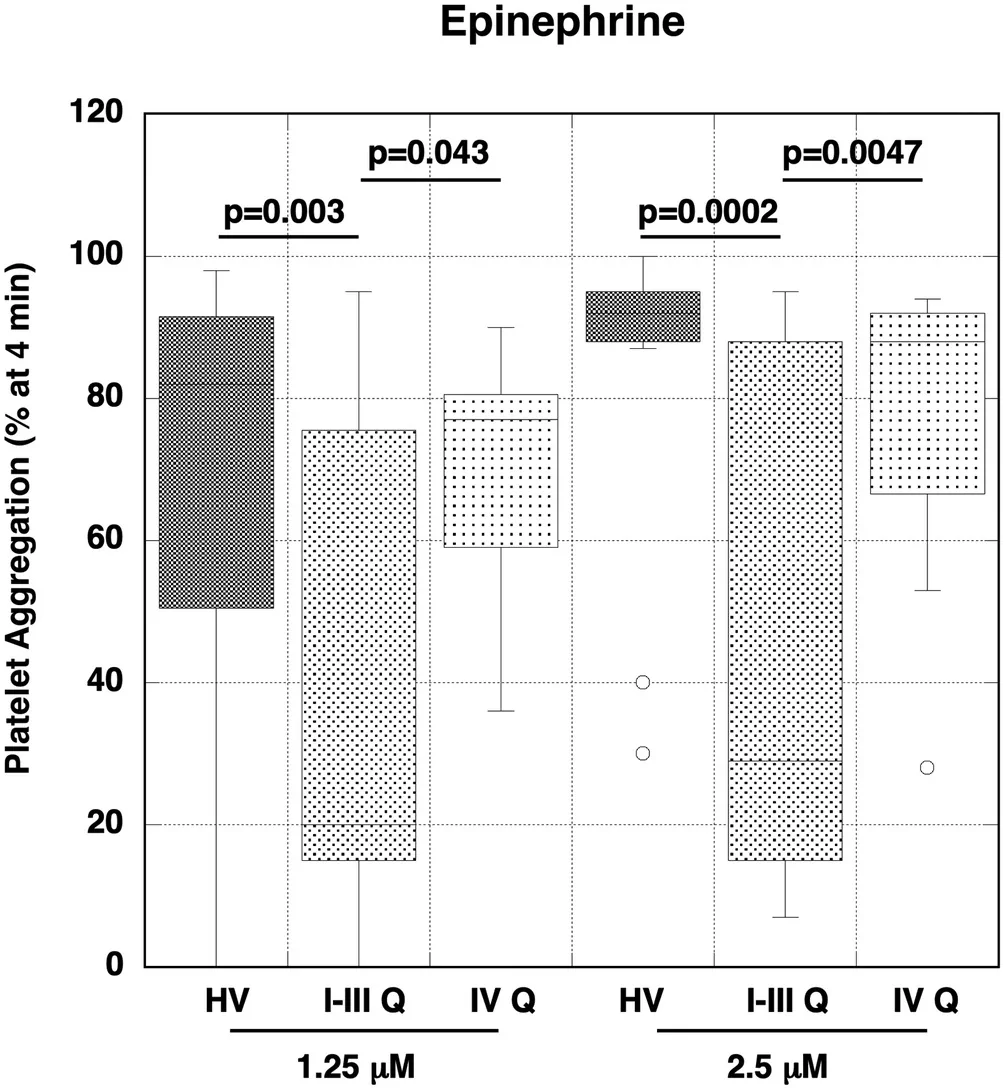
Assessment of platelet aggregation induced by epinephrine between healthy volunteers and patients with migraine divided by lesion load. On the left side, the comparison of platelet aggregation induced by epinephrine at the concentration of 1.25 μM among healthy volunteers (HV—dark gray box) and patients with migraine divided by lesion load: first to third quartile of lesion load (< 146.25 mm^3^ or no lesions) (light gray box) and fourth quartile of lesion load (≥ 146.25 mm^3^) (white box). On the right side, the same comparison of platelet aggregation induced by epinephrine at the concentration of 2.5 μM. The median value and 25th to 75th percentiles are reported. *p* values are reported when significant.

No significant difference was detected in platelet aggregation in response to Collagen at 1.25 μM between the two populations, while platelet aggregation in response to Collagen at 2.5 μM was higher in migraine patients compared to healthy volunteers (*p* < 0.020) (Figure 3).

**FIGURE 3:**
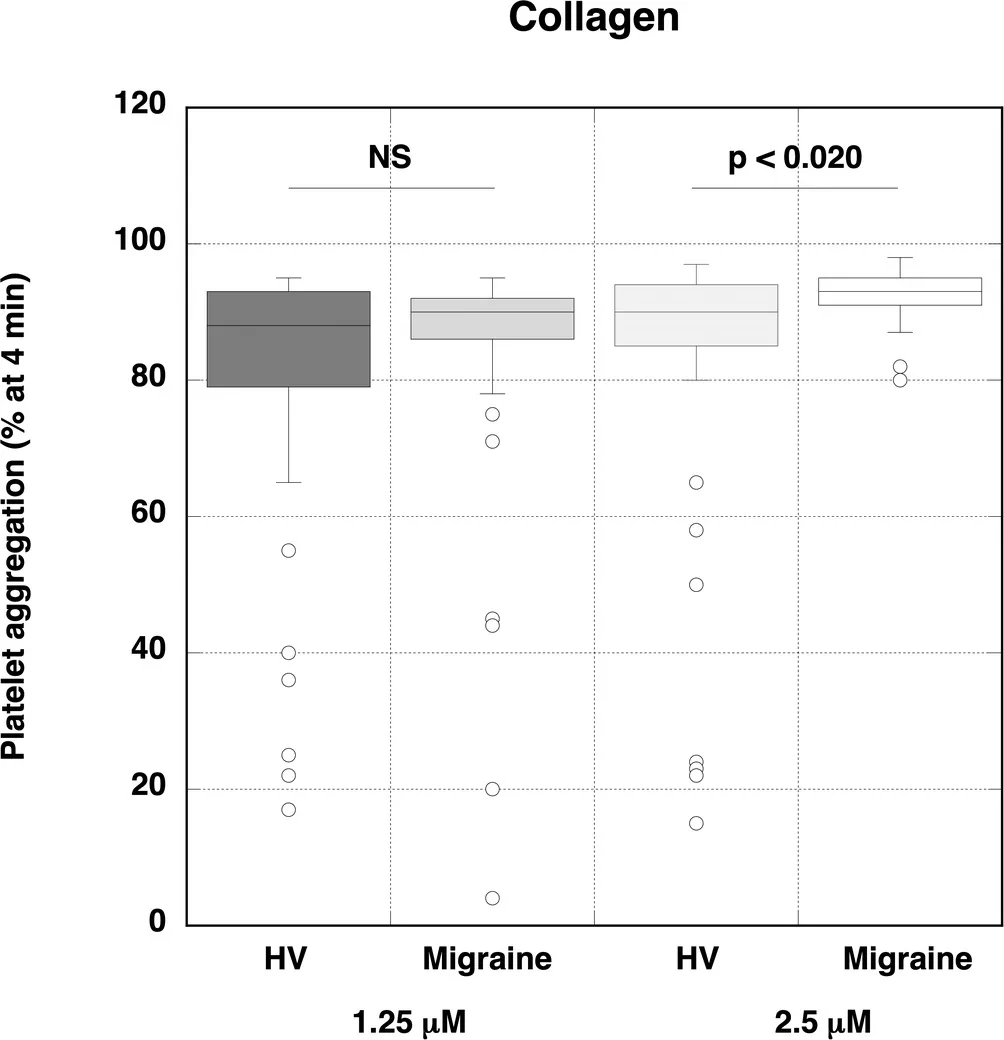
Assessment of platelet aggregation induced by Collagen between healthy volunteers and patients with migraine. On the left side, the comparison of platelet aggregation induced by Collagen at the concentration of 1.25 μg/mL between healthy volunteers (HV) and patients with migraine (Migraine). On the right side, the comparison of platelet aggregation induced by Collagen at the concentration of 2.5 μg/mL between HV and patients with migraine. The median value and 25th to 75th percentiles are reported. *p* values are reported when significant.

No significant differences were detected in platelet aggregation in response to two different concentrations of ADP (1.25 and 2.5 μM) and arachidonic acid between the two populations (Figures 4 and 5).

**FIGURE 4:**
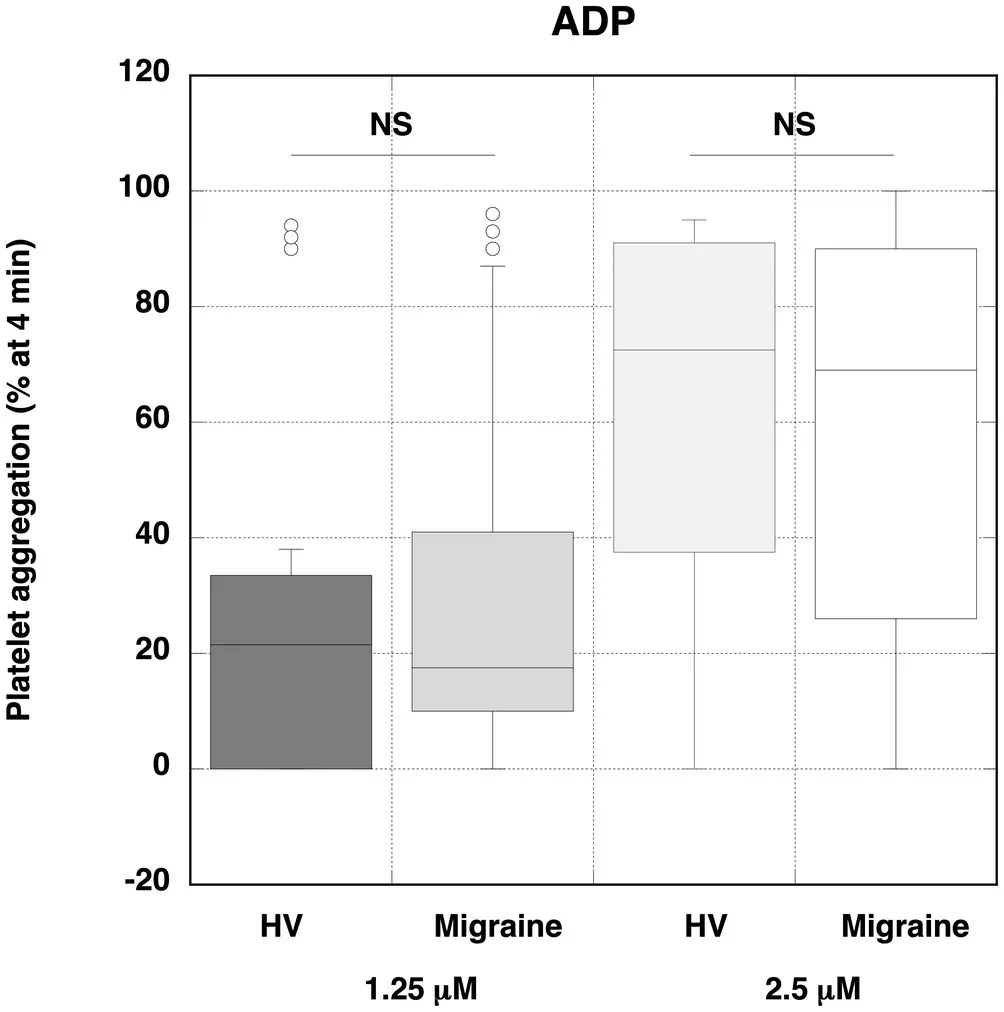
Assessment of platelet aggregation induced by ADP between healthy volunteers and patients with migraine. On the left side, the comparison of platelet aggregation induced by ADP at the concentration of 1.25 μM between healthy volunteers (HV) and patients with migraine (Mig). On the right side, the comparison of platelet aggregation induced by ADP at the concentration of 2.5 μM between healthy volunteers (HV) and patients with migraine (Mig). The median value and 25th to 75th percentiles are reported. *p* values are reported when significant.

**FIGURE 5:**
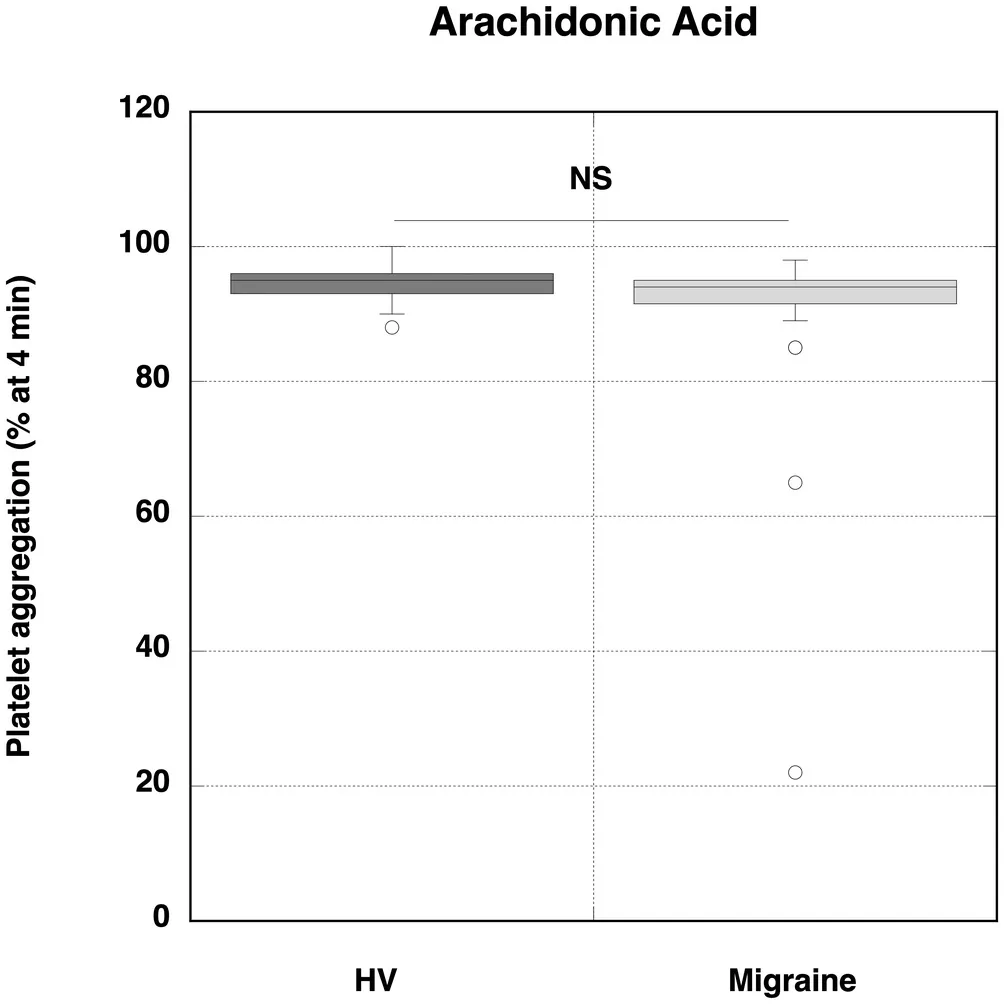
Assessment of platelet aggregation induced by arachidonic acid between healthy volunteers and patients with migraine. On the left side, the comparison of platelet aggregation induced by arachidonic acid at the concentration of 0.5 mM between healthy volunteers (HV—left side) and patients with migraine (right side). The median value and 25th to 75th percentiles are reported. *p* values are reported when significant.

Within the migraine cohort, we observed no statistically significant differences in platelet aggregation between MA and MO across agonists (epinephrine, collagen, ADP, and arachidonic acid; Table S1). There was, however, a non‐significant trend toward higher aggregation with epinephrine 1.25 μM in MA versus MO (median [IQR]: 22 [23–90] vs. 16 [14–77]; *p* = 0.0602), which may reflect the limited sample size.

## 4 Discussion

Our proof‐of‐concept study presents novel findings, demonstrating for the first time that, in a population of patients with migraine largely—though not entirely—free of vascular risk factors, compared to healthy volunteers: (i) platelet aggregation in response to epinephrine at different concentrations was reduced; (ii) among migraine sufferers, those with a higher load of WMLs exhibited increased platelet aggregation in response to epinephrine at different concentrations compared to those with a smaller lesion load or no lesions; and (iii) patients with a smaller lesion load or no lesions presented a lower platelet aggregation in response to epinephrine at different concentrations than healthy volunteers.

Several important considerations must be addressed to interpret our findings. Firstly, the mechanisms underlying the development of cerebral lesions in migraine patients are not fully understood, and histopathological data on WMLs in migraine are lacking. Both the duration of the disease and the frequency of migraine attacks are believed to influence lesion development, and comorbidities may also contribute. Various attack‐related mechanisms have been proposed to explain the formation of WMLs, including repeated intracerebral hemodynamic changes such as oligemia and focal hypoperfusion leading to tissue ischemia. In MA, recurrent hemodynamic fluctuations are particularly relevant, as they have been implicated in the pathogenesis of ischemic events. Patients with MA may exhibit impaired vasomotor reactivity to hypercapnia and subtle deficits in cerebral autoregulation compared to those with MO, as demonstrated by Vernieri et al. [25]. These cerebrovascular abnormalities, when present, may contribute to the increased risk of ischemic stroke observed in this subgroup. The radiological characteristics of these MRI‐detected hyperintensities resemble those seen in chronic WMLs of ischemic origin, and histopathological findings in elderly patients with WMLs suggest impaired vascular and blood–brain barrier integrity, further supporting the theory that WMLs in migraine may result from microvascular ischemic injury. While WMLs in the elderly with hypertension are predominantly periventricular [26, 27, 28, 29], migraine is more often associated with deep WMLs [9, 12], which can be detected even in childhood migraine patients. Indeed, WMLs are present in 10% of pediatric migraine patients [29], suggesting they occur early in life and remain stable over time. However, at least two longitudinal MRI studies have shown that silent WMLs may progress over time (3 and 9 years later) [11, 12], with some cases showing occasional regression. A lower baseline frequency of migraine attacks has been associated with a reduction in WMLs over a 3‐year period, indicating a potential long‐term relationship between migraine and WMLs.

Secondly, large‐scale population studies [15, 30, 31, 32] have shown an increased risk of ischemic and haemorrhagic stroke in migraine patients, particularly in otherwise healthy women with MA under 45 years [32]. A meta‐analysis of 16 cohort studies involving over one million individuals and a 26‐year follow‐up confirmed that migraine is a risk factor for stroke (adjusted hazard ratio [aHR] 1.42) and MI (aHR 1.4), with the presence of aura further increasing stroke risk (aHR 1.56) and all‐cause mortality (aHR 1.20). Surprisingly, no gender differences were noted in the meta‐analysis [33].

Thirdly, although the precise etiology of cerebral lesions in migraine patients remains unclear, a micro‐ischemic mechanism is suspected. Platelets play a role in the development of ischemia through thromboembolism and ensuing hypoperfusion. Previous research has shown that migraine patients exhibit increased platelet activation and aggregation during and between attacks, independent of migraine subtype. Elevated levels of platelet‐activating factor [15], urinary 11‐dehydrothromboxane B2 [16], platelet surface P‐selectin and platelet‐leukocyte aggregates [17] have been observed in migraine patients during the interictal period, suggesting a role in sterile brain inflammation and pain signaling by promoting the release of proinflammatory cytokines. Further supporting this evidence, low‐dose aspirin prophylaxis (80 mg daily) has been shown to reduce the frequency of MA [18] in patients without PFO [19]. The more recent LEARNER study also demonstrated that platelet procoagulant potential in MA patients, which was higher than in HV, normalized following PFO closure. MA patients not on aspirin exhibited platelet aggregation in response to low concentrations of thrombin that were greater than those observed in patients before PFO closure, and they also had a higher number of circulating tissue factor (TF)‐positive platelets compared with MA patients on aspirin. These data suggest that the mechanisms leading to agonist‐induced platelet aggregation are regulated by aspirin, which also partially controls platelet‐associated TF expression [34]. Additionally, high on‐aspirin platelet reactivity, better known as aspirin resistance, has been reported more frequently in patients with migraine than in healthy individuals or patients with coronary artery disease taking aspirin 325 mg [33]. Interestingly, one study found that oxidative stress reduced platelet aggregation responses to ADP and collagen in MA patients compared to HV during the interictal period, suggesting that the “in vitro” anomalous platelet behavior in MA patients might be considered the transient expression of exhausted platelets to “in vivo” stimulation and probably related to an increased vulnerability to oxidative stress [35]. Moreover, as highlighted by Paolucci et al. [36], oxidative stress may play a pivotal role in promoting endothelial dysfunction, which is increasingly recognized as a key mechanistic link between migraine with aura and ischemic stroke, through pathways involving nitric oxide depletion, vascular inflammation, and impaired cerebrovascular reactivity.

The strengths of our study include: (i) the prospective nature of data collection, (ii) the strict radiological and clinical inclusion criteria, and (iii) the blinded neuroimaging assessments conducted by the same neuroradiologist to avoid diagnostic bias. However, the main limitations of our study are the small sample size, due to our strict enrolment criteria, and the inclusion of both patients with MA and MO. Nevertheless, the study was designed to assess platelet aggregation based on the presence and extent of WMLs, regardless of the migraine subtype. Recognizing that migraine phenotype (with vs. without aura) may modulate platelet behavior, we conducted a phenotype‐stratified analysis and found no differences in platelet aggregation between MA and MO across agonists. However, we observed a non‐significant tendency toward greater epinephrine‐induced aggregation at 1.25 μM in MA versus MO (median [IQR]: 22 [23–90] vs. 16 [14–77]; *p* = 0.0602), an effect likely underpowered given the limited sample size. Larger, prospectively stratified cohorts are warranted to clarify this finding. No further subanalysis was feasible for the subgroup with vascular factors because of subgroup imbalance and limited statistical power.

Drawing upon prior data available in the literature, we had expected to find an increased platelet aggregation in migraine patients compared to controls. Unexpectedly, our findings revealed reduced platelet aggregation in the migraine cohort—specifically, a blunted response to epinephrine, whereas aggregation induced by ADP, collagen, and arachidonic acid remained comparable between groups—with notable differences observed based on lesion load identified via MRI. Mechanistically, epinephrine acts through platelet α₂A‐adrenergic receptors; interictal up‐regulation of nitric oxide/cGMP signaling in migraine can dampen this pathway [37], whereas chronic endothelial stress and elevated catecholamine tone in patients with a higher WML burden may resensitize it [38]. This reduced platelet aggregation might represent a protective mechanism, potentially functioning as an adaptive response to frequent metabolic and vascular stress associated with migraine. This hypothesis aligns with the paradigm of ischemic preconditioning, whereby repeated sub‐threshold ischemic episodes—analogous to the alternating oligaemic–hyperaemic waves generated by cortical spreading depression during migraine aura—elicit genomic, metabolic, and endothelial adaptations that enhance cerebrovascular tolerance to subsequent ischemic insults [39]. This mechanism would be less effective in patients with a higher lesion load—as reflected by a partial rebound of epinephrine responsiveness—irrespective of disease duration, given the cross‐sectional design of the study and the absence of clinical differences between migraine patients with low and high lesion loads. This subset of patients might thus be at greater risk of developing ischemic damage. As noted by Bigal, if migraine is indeed a risk factor for silent brain lesions, antiplatelet therapy could play a more pivotal role among high‐risk groups in preventing silent infarcts [40], rather than focusing on mere migraine prevention. While low‐dose aspirin may afford a slight reduction in migraine frequency [41], its foremost benefit could lie in mitigating the risk of silent infarcts, rather than alleviating current pain and disability.

From this perspective, the results of our study carry potential translational relevance. Assessing platelet aggregation might serve as a valuable marker of cerebrovascular risk, helping to identify a high‐risk subgroup among migraine patients. However, given the exploratory nature of our findings, further research is needed to determine whether ischemic mechanisms contribute to the development of WMLs and to clarify the role of platelet aggregation in this context.

## 5 Conclusions

This proof‐of‐concept study demonstrates that migraine is associated with reduced platelet responsiveness to aggregation stimuli compared to HV, suggesting a potential protective mechanism for the brain. Notably, the same mechanism was not observed in patients with a higher burden of WMLs, whose platelet aggregation levels were comparable to those of HV, indicating a possible ischemic pathophysiology linked to WMLs in migraine patients. This study carries translational relevance, as assessing platelet aggregation could help identify a subset of migraine patients at higher risk for cerebrovascular events. Further studies are warranted to confirm these results.

## Author Contributions

I.M., F.P. and V.D.P. conceived the study. I.M., M.P., M.A. and M.T. enrolled the patients and collected the clinical data. C.G. analyzed the neuroradiological data. M.C. and F.P. performed platelet aggregation. I.M., M.C. and F.P. set up the database and analyzed the data. I.M. and F.P. performed statistical analyses. The first draft of the manuscript was written by I.M., V.D.P. and F.P. and all authors commented on previous versions of the manuscript. All authors were involved in revising the manuscript for intellectual content. All authors read and approved the final manuscript.

## Funding

The authors have nothing to report.

## Ethics Statement

All participants provided written informed consent. The study was approved by the Ethics Committee of Human Experimentation of Policlinico Umberto I University Hospital (Protocol No. 152/18 ce ref. 4839) and complied with the latest version of the Declaration of Helsinki.

## Consent

Patients gave their written consent to the anonymous scientific publication of their data.

## Conflicts of Interest

The authors declare no conflicts of interest.

## Supporting information


**Figure S1:** Periventricular white matter lesions. T2‐TSE (Turbo Spin Echo) axial images (A) and FLAIR (Fluid Attenuated Inversion Recovery) axial images (B). Arrows indicate periventricular white matter lesions (hyperintensities compared to normal appearing white matter). R: right; L: left.

**Figure S2:** Deep white matter lesions. T2‐TSE (Turbo Spin Echo) axial images (A, C) and FLAIR (Fluid Attenuated Inversion Recovery) axial images (B, D). White arrows indicate subcortical white matter lesions and blue arrows indicate deep white matter lesions (hyperintensities compared to normal appearing white matter) R: right; L: left.

**Table S1:** Comparison of platelet aggregation induced by different agonist between patients with migraine with (MA) and without aura (MO).

## Data Availability

The datasets generated or analyzed during the current study are available from the corresponding author upon reasonable request.
