# Supplementary material for: Subclinical Cerebral Lesions in Migraine: A Proof‐of‐Concept Study on Platelet Aggregation
Source: Eur J Neurol. 2026 Nov 2;33(11):e70432. doi: 10.1111/ene.70432 (PMC13726475; doi:10.1111/ene.70432)
Supplement: Supplementary file 1 [file ENE-33-e70432-s002.docx]

**Supplemental table 1. Comparison of Platelet Aggregation induced by different agonist between patients with migraine with (MA) and without aura (MO)**

| **Agonist** | **MO** | **MA** | **p value** |
| --- | --- | --- | --- |
| Epinephrine 1.25 μM | 16 (14 – 77) | 22 (23 – 90) | 0.0602 |
| Epinephrine 2.5 μM | 26 (15 – 53) | 39 (16-88) | 0.2584 |
| Collagen 1.25 μg/ml | 24 (70 – 92) | 22 (88 – 92) | 0.2608 |
| Collagen 2.5 μg/ml | 25 (87 – 94) | 22 (91 – 95) | 0.1230 |
| ADP 1.25 μM | 12.5 (5 -43) | 20 (11-28) | 0.4874 |
| ADP 2.5 μM | 47.5 (12 – 92) | 69 (22 – 89) | 1 |
| Arachidonic Acid 0.5 mM | 94 (91 – 95) | 94 (92 – 95) | 0.9300 |

The median value and 25th to 75th percentiles are reported.
